# Ileal Biofilms Confirm SIBO–IBS Hypothesis: Comment on PMID 38798120

**DOI:** 10.1111/nmo.70183

**Published:** 2025-10-24

**Authors:** Maximilian Baumgartner, Christoph Gasche

**Affiliations:** ^1^ CeMM Research Center for Molecular Medicine of the Austrian Academy of Sciences Vienna Austria; ^2^ Division of Gastroenterology and Hepatology, Department of Internal Medicine III Medical University of Vienna Vienna Austria

**Keywords:** bile acid malabsorption, functional gastrointestinal disorders, irritable bowel syndrome, mucosal biofilms, small intestinal bowel overgrowth

We share some concerns by Dr. Kashyap et al. [1] on the rampant use of the lactulose hydrogen breath test (LHBT) for the diagnosis of small intestinal bacterial overgrowth (SIBO). However, the SIBO–IBS hypothesis should not be completely dismissed as a potential pathomechanism in a subset of patients with disorders of gut–brain interaction.

We previously reported mucosal biofilms in the terminal ileum and colon in two‐thirds of IBS patients. Such biofilms represent an overgrowth of bacteria invading the mucus layer [2]. We validated increased bacterial concentrations by molecular (qPCR), histologic (DAPI, FISH), and electron microscopic analysis. As per definition, biofilms in the terminal ileum represent a type of SIBO, which we like to term lower SIBO (in contrast to upper SIBO with bacterial overgrowth in the duodenum and proximal jejunum). Direct visualization of lower SIBO by endoscopy and histology is far less disputable than any measurement of breath test or radionucleotide transit time [3]. The terminal ileum is a key metabolic hub involved in regulating bile acid homeostasis. Indeed, the increase of microbial biomass was associated with altered microbial bile acid metabolism and malabsorption [4], which could explain common IBS symptoms such as diarrhea and pain. A biofilm protects persister cells from antibiotics, explaining the temporary, non‐sustained clinical response commonly followed by repeated antibiotic cycles in IBS [5]. These biofilms also consist of polymicrobial communities, including archaea and fungi [6], representing a microbial strategy to better withstand the harsh environment of the small intestine.

More recently, we found ileal biofilms in 24 out of 40 IBS‐D or IBS‐M patients (60%). LHBT was positive in 95% of biofilm‐positive patients vs. 54% of biofilm‐negative patients (Table 1, *p* < 0.01 Fisher's exact test). In contrast, one out of 24 biofilm‐positive patients was positive in the glucose hydrogen breath test (GHBT). Thus, lower SIBO (i.e., the presence of ileal biofilms) is highly prevalent in IBS‐D and IBS‐M, associated with positive LHBT (but not GHBT), in agreement with the SIBO‐IBS hypothesis. GHBT could not identify patients with lower SIBO.

**TABLE 1:** Association of SIBO‐test and endoscopically visible mucosal biofilms in the ileum of IBS‐D/M patients as defined in (Baumgartner et al., Gastroenterology [2]).

SIBO‐test		Biofilm‐positive	Biofilm‐negative	Fisher's exact
GHBT	Positive	1 (4%)	2 (12%)	n.s.
	Negative	23 (96%)	14 (88%)	
LHBT	Positive	23 (95%)	9 (54%)	*p* < 0.01
	Negative	1 (5%)	7 (46%)	

Abbreviations: GHBT, glucose hydrogen breath test; LHBT, lactulose hydrogen breath test.

For the reasons of non‐penetrable biofilms, polymicrobial communities, and non‐sustainable clinical response, we concur with Dr. Kashyap and colleagues on the injudicious use of antibiotics. Repeated antibiotic cycles select for resistant bacterial traits, lead to a further decrease of diversity, and ultimately perpetuate the underlying problem of western lifestyle‐associated GI disorders. An alternative approach, such as endoscopic removal of biofilms using biofilm‐disrupting liquids, is currently under investigation [7].

## Author Contributions

**Maximilian Baumgartner:** conceptualization (equal); writing – original draft (equal); formal analysis (lead); writing – review and editing (equal). **Christoph Gasche, Maximilian Baumgartner:** conceptualization (equal); writing – original draft (equal); formal analysis (lead); writing – review and editing (equal), securing funding (lead).

## Conflicts of Interest

The authors declare no conflicts of interest.

## Linked Articles

Critical appraisal of the SIBO hypothesis and breath testing: A clinical practice update endorsed by the European society of neurogastroenterology and motility (ESNM) and the American neurogastroenterology and motility society (ANMS), https://doi.org/10.1111/nmo.14817.
